# The Mechanisms of Psychedelic Visionary Experiences: Hypotheses from Evolutionary Psychology

**DOI:** 10.3389/fnins.2017.00539

**Published:** 2017-09-28

**Authors:** Michael J. Winkelman

**Affiliations:** School of Human Evolution and Social Change, Arizona State University, Tempe, AZ, United States

**Keywords:** psychedelic, cognition, mysticism, shaman, consciousness, neurophenomenology, mirror neuron system

## Abstract

Neuropharmacological effects of psychedelics have profound cognitive, emotional, and social effects that inspired the development of cultures and religions worldwide. Findings that psychedelics objectively and reliably produce mystical experiences press the question of the neuropharmacological mechanisms by which these highly significant experiences are produced by exogenous neurotransmitter analogs. Humans have a long evolutionary relationship with psychedelics, a consequence of psychedelics' selective effects for human cognitive abilities, exemplified in the information rich visionary experiences. Objective evidence that psychedelics produce classic mystical experiences, coupled with the finding that hallucinatory experiences can be induced by many non-drug mechanisms, illustrates the need for a common model of visionary effects. Several models implicate disturbances of normal regulatory processes in the brain as the underlying mechanisms responsible for the similarities of visionary experiences produced by psychedelic and other methods for altering consciousness. Similarities in psychedelic-induced visionary experiences and those produced by practices such as meditation and hypnosis and pathological conditions such as epilepsy indicate the need for a general model explaining visionary experiences. Common mechanisms underlying diverse alterations of consciousness involve the disruption of normal functions of the prefrontal cortex and default mode network (DMN). This interruption of ordinary control mechanisms allows for the release of thalamic and other lower brain discharges that stimulate a visual information representation system and release the effects of innate cognitive functions and operators. Converging forms of evidence support the hypothesis that the source of psychedelic experiences involves the emergence of these innate cognitive processes of lower brain systems, with visionary experiences resulting from the activation of innate processes based in the mirror neuron system (MNS).

## Introduction

Institutionalized use of psychedelics in religions of pre-modern societies worldwide reveal the central roles of these substances in the evolution of spiritual experiences, cultures, and religions (Schultes et al., 1992; Rätsch, 2005; Rush, 2013; Ellens, 2014; Winkelman, 2014; Winkelman and Hoffman, 2015; Panda et al., 2016). The role of psychedelics in human evolution is indicated by evidence that psychedelics bind to human serotonergic receptors with a higher affinity than they do to those receptor systems in other primates (Pregenzer et al., 1997). The reasons for the roles of psychedelics in cultural evolution are revealed by neuropharmacological research on psychedelic effects on brain processes. The interaction of psychedelics with the innate structures of the human brain produces novel forms of information and integrative cognitive processes (Carhart-Harris et al., 2014b; Froese, 2015; Gallimore, 2015). This suggests that psychedelic substances operated as environmental factors selecting for an enhanced capacity for specific forms of information processing.

The neuropharmacological dynamics of psychedelics are central to understanding the nature of spiritual experiences. Psychedelics are associated with pre-modern religious forms and the early history of the current major world religions (Winkelman and Hoffman, 2015). Furthermore, there is evidence established by double blind clinical studies that spiritual experiences are directly caused by neuropharmacological effects of psychedelic substances (Griffiths et al., 2006, 2008, 2011). Why should the pharmacological effects of psychedelics so consistently produce such significant experiences? What neuropharmacological mechanisms produce these powerful visionary experiences?

While neuropharmacology is conventionally conceptualized as the study of the effects of drugs on behavior, the ultimate goal must also offer some explanation of how drugs have effects on people's experiences. Why do psychedelics produce the kinds of experiences that lead to the foundation of cultures and religions? How do the pharmacological effects so reliably and quickly produce the kinds of experiences that mystical devotees spend a lifetime pursuing for just a glimpse of these alleged eternal truths?

Some might consider the answers to these questions to be beyond the purview of neuropharmacology, perhaps best left to philosophers. But a mature neuropharmacology ought to be able to offer a cogent explanation of how it is that the neurochemistry of an exogenous compound produces activity and functional modifications in the brain that lead people across time and place to report experiences of a profoundly spiritual nature, often with great significance to the individual and even society.

A neuropharmacological explanation of psychedelic effects on human experience can be found in the approach of neurophenomenology, “a research programme aimed at bridging the explanatory gap between first-person subjective experience and neurophysiological third-person data, through an embodied and enactive approach to the biology of consciousness…{N}europhenomenology is then viewed as a novel scientific method building on a corpus of intersubjectively-invariant first-person reports that may broaden the horizon of objective science” (Khachouf et al., 2013, p. 1). We need to know what is being experienced in order to identify what it is we are ultimately trying to explain.

Neurophenomenology is an approach to the understanding of the structure and content of phenomenal experience in terms of principles operating at the neurological level (see Laughlin et al., 1992; Varela, 1996; Thompson and Varela, 2001; Thompson, 2007). Or in other terms, the first person perspectives of personal experience are explained by reference to some homologous causal features identified by third person perspectives on brain operation. If what people experience ought to be explained in terms of pharmacological actions, then psychedelics are an excellent example of this challenge presented to neuropharmacology.

The experiences to be explained are not just some debatable philosophical construct but an objective domain of experience revealed by empirical evidence. A variety of research projects provide converging findings that confirm the empirical nature of the domains of altered states of consciousness (see Preller and Vollenweider, 2016 for review). These domains of human experience are assessed through psychometric instruments such as: the Altered State of Consciousness Questionnaire (Dittrich et al., 1985; Dittrich, 1998), later modified as the APZ-OAV (Dittrich, 1998); the Phenomenology of Consciousness Inventory (Pekala et al., 1986); the Hallucinogen Rating Scale (Strassman et al., 1994; Riba et al., 2001); the Mysticism scale (Hood et al., 2001); the Mystical Experiences Questionnaire (MacLean et al., 2012; Barrett et al., 2015); and the Five Dimensions of Altered States of Consciousness (5D-ASC; Studerus et al., 2010). These studies provide empirical evidence of common core dimensions to experiences of pharmacologically and non-pharmacologically induced alterations of consciousness.

If the ultimate goal neuropharmacology includes an explanation of the nature of first-person psychedelic experiences in terms of pharmacological, neurological and functional brain mechanisms, what is this experience to be explained? What is the nature of psychedelic experiences? There are number forms of psychedelic experience, exemplified in differences in mystical and shamanic psychedelic experiences described below. The empirical data regarding these experiences are startling and perhaps even confusing for the following reasons:

1. Psychedelics reliably elicit experiences that are virtually indistinguishable from mystical experiences induced through prolonged austerities and disciplined contemplative practice (Smith, 2000; Richards, 2016; Yaden et al., 2017); and
2. In contrast to the mystical features of psychedelic-induced experiences, psychedelic use in pre-modern shamanic cultures produced a distinctive worldview characterized by animism, an experience of transforming into an animal, and the perspective of entheogens, viewing these substances as generating experiences of spiritual entities within the person and environment (Dobkin de Rios, 1984; Winkelman, 2013b).

The differences in psychedelic-induced mystical and shamanic experiences illustrate that while these substances reliably produce certain kinds of experience, the forms of experience may vary considerably—one agent, variable experiences. Secondly, the similarity of psychedelic and non-psychedelic mystical experiences suggests that the explanation of psychedelic experiences is not through mechanisms unique to psychedelics, but rather through shared mechanisms affected by non-drug procedures.

### Hypotheses

This leads to the central hypotheses of this paper:

1. The effects of psychedelics in producing visionary experiences involve the same mechanisms elicited by other non-drug mechanisms for altering consciousness and producing visionary experience; and
2. These mechanisms involve a dis-inhibition of regulatory mechanisms of the brain that releases a number of innate modules, operators or intelligences, especially the mirror neuron system (MNS).

Perhaps the most challenging fact that requires explanation in neuropharmacological terms is how psychedelic-like visionary experiences can also occur endogenously as a consequence of a variety of ritual behaviors, physical activities, and pathophysiological conditions (Winkelman, 2010a, 2013a). What are the mechanisms by which pharmacological, mental, behavioral and organic processes produce what appears to be the same kind of experience?

Concepts of innate intelligences, operators and modules identified by Gardner (1983, 2000; also see d'Aquili and Newberg, 1999; Boyer, 2001; Ernandes, 2013) are used in the cognitive science of religion to explain the universality of supernatural beliefs and experiences. Supernatural experiences are also central features of psychedelic visionary experiences in societies worldwide. Parsimony suggests a common biological bases for the supernatural beliefs found worldwide and the supernatural beliefs stimulated by psychedelics.

Several models propose that similar mechanisms are shared by many different alterations of consciousness (Mandell, 1980; Dietrich, 2003; Vaitl et al., 2005; Winkelman, 2011). Common mechanisms that psychedelics share with other alterations of consciousness involving effects that disable the prefrontal cortex and default mode networks (DMNs). These commonalities in the mechanisms by which psychedelics and other processes alter consciousness provide a bridge between neuronal and behavioral pharmacology. This leads to the hypotheses regarding the common mechanisms by which psychedelic and various procedures (meditation, hypnosis) induced visionary experiences. This involves the consequences of compromising the pre-frontal cortex and DMN, leading to the release of innate modular brain operators and functions. One of these, the MNS, is hypothesized to produce visionary experiences. This system was central in the evolution of human cognitive and symbolic capacities of imitation, mimesis, and symbolic representation.

## Psychedelics as elicitors of spiritual experience

Some of the psychedelic-induced experiences that require explanation in neurophenomenological terms are revealed by Griffiths et al. (2006, 2008) who provided empirical evidence that psilocybin induces a variety of features of classic mystical experiences (Stace, 1960) under double-blind conditions. Comparison with control periods showed psilocybin produced significantly higher ratings on both introvertive and extrovertive mystical experiences, an internal identification of the divine within oneself, or an identification with an external divinity, losing all sense of self-identity in a unity with the cosmos. Psilocybin also induces a perception of sacredness and a direct intuitive knowledge characterized as ineffable; a sense of transcendence of time and space; experiences of oceanic boundlessness; and positive mood, as evidenced by self-reports of significantly higher levels of peace, harmony, joy, and happiness. In addition, there were persisting effects of positive mood changes and altruistic social behaviors noted by third-party observers (Griffiths et al., 2008; MacLean et al., 2011). Follow-up studies demonstrated persisting changes in the personality trait of openness, as well as the personal and spiritual meaning that participants gained from the experience (Griffiths et al., 2011).

The intrinsic mystical effects of psychedelics were further confirmed in studies with LSD (Liechti et al., 2016). While the authors reported that their LSD-induced mystical experiences appeared to be less intense than those reported in the psilocybin studies, they note that this may be the result of set and setting differences. The likelihood of more spiritually inclined participants in the Griffiths studies, along with their more extensive preparation, may account for the higher levels of psilocybin-induced mystical experiences (Polito et al., 2010; but see Ray, 2010, since psilocybin's active ingredient psilocin acts differently than LSD at a variety of receptor sites).

Psychedelics induce experiences that alter the sense of knowing through an experience of dissolution of the self (Richards, 2016). The experiences induced by psychedelics include the sense that knowledge is ineffable, beyond explanation, yet somehow comprehensible through some direct unity with this knowledge. The comparison of mystical experiences induced by psychedelics with those induced by mental practice found the former to be significantly more intensely mystical, having more of a positive or existential impact on their life, and having given the person an increased sense of purpose and spirituality (Yaden et al., 2017). Psychedelics make easily accessible and more intense what are generally rare mystical experiences.

Why do psychedelics evoke what are arguably among the most important spiritual experiences of humanity? This answer lies in the relationship of psychedelics to the evolution of human cognitive and social capacities. This relationship derived from the pharmacological effects on the brain of the substances in the environment which were incorporated into religious rituals across the early prehistory of humanity because of specific adaptive effects enhancing certain aspects of human cognition and social behavior.

### Evolutionary adaptations to plant toxins

Humans have a long-term symbiotic relationship with psychedelics, derived from selective influences exercised by these environmental factors (Sullivan et al., 2008); this effect derives from the functions of plant substances as exogenous analogous of human neurotransmitters (Sullivan and Hagen, 2002). Psychedelics affect a variety of neurotransmitters, including serotonin, acetylcholine, norepinephrine, dopamine and many others (Passie et al., 2008; Hintzen and Passie, 2010; Ray, 2010).

Evidence for an influence of psychedelics on hominin evolution is found in the much higher sensitivity of the human serotonergic system for bonding with psychedelics than is the case of great apes (Pregenzer et al., 1997). Human and chimpanzee 5-HT_1D_ receptor amino acid sequences differ in ways that indicate molecular divergences in human evolution. Human serotonergic systems (as compared to chimpanzees) have a significantly greater binding with LSD and the ergots metergoline and dihydroergotamine (Pregenzer et al., 1997). These differences indicate that psychedelic plant substances exercised selective effects that contributed to the evolution of humanity's neuropsychology. The inevitability of psychedelic effects on human evolution is attested to by the worldwide distribution and cultural use of psilocybin-containing mushrooms (Guzman et al., 1998; Froese et al., 2016).

Evolutionary paradigms suggest that fitness benefits accrued to our ancestors as a consequence of their ability to utilize psychoactive substances to enhance the operation of neurotransmitter systems. Sullivan and Hagen (2002) proposed that the evidence for relationships of psychotropic plant substances with humans' cognitive capacities indicate that there were selective benefits derived from substance use. The fitness advantages that psychedelic substances offered impacted the evolution of human consciousness and aspects of human social psychology (Winkelman, 2013b). Smith (1999) proposed that evolutionary influences of diverse classes of plant substances include enhanced vigilance, pain management, increased mating opportunities, reduction of apprehension and stress, feelings of euphoria, increased endurance and self-confidence, enhanced sensory and mental acuity, reduction of defensiveness, and reduction of depression and self-defeating activities.

The effects of plant substances on the brain's reward centers display hallmark features of natural adaptations (Smith and Tasnadi, 2007). These resulted from selective pressures for human metabolic systems that were capable of using these exogenous sources of neurotransmitter precursors that were designed to function as toxins to deter consumption of the plant. There was a positive selection for pituitary cyclase-activating polypeptide precursor (PACAP), a uniquely human feature that emerged after separation from our common ancestor with chimpanzees (Wang et al., 2005; also see Rockman et al., 2005). The PACAP precursor gene was a consequence of positive selection for enhancing the biological activity of neuropeptides, protecting them from enzymatic degradation and increasing their affinity for receptor binding (Wang et al., 2005). Sullivan et al. (2008) pointed out that the mammalian xenobiotic-metabolizing cytochrome P450 provides evidence of a deep evolutionary history of adaptation to plant toxins resulting in a positive selection for the CYP2D enzyme that enables the body to metabolize plant toxins.

### Shamanism: the ritual context of selection for psychedelic uses

The selective influences of psychedelics on human adaptation occurred within the context of social rituals. This fundamental role of ritual reflects the social importance of collective rituals in primates—communication for enhancing cooperation within groups (see Laughlin and d'Aquili, 1974; d'Aquili et al., 1979)—which was expanded during hominin evolution (Winkelman, 2009, 2015). Ritual behaviors provided the most significant collective institutions of early modern human societies (Rossano, 2009, 2012, 2015). These ritual practices gave rise to the practices of shamanism found in pre-modern societies worldwide (Winkelman, 2002, 2010a,b; Rossano, 2011).

Shamanic ritual was the core area of social life in the selection for enhancement of capacities stimulated by psychedelics. This included the stimulation of ritual healing responses (Winkelman, 2009, 2010b), such as hypnotic susceptibility and placebo effects (McClenon, 1997, 2002; Rossano, 2009, 2011). Furthermore, there is abundant evidence of multiple therapeutic mechanisms elicited by psychedelics (Winkelman and Roberts, 2007a,b). Psychedelic substances enhanced the experiences at the core of shamanic practices and extended the psychological, cognitive and social potentials of ritual alterations of consciousness (Winkelman, 2013b; also see Bousoa et al., 2015).

A central feature of shamanic ritual is divination, the acquisition of information manifested in visionary experiences. Ritual activities (fasting, drumming, dancing, chanting) produce habituating effects on the brain's information processing system (Winkelman, 2010a). Profound alterations of the brain's ordinary routines results in a greater randomization of brain activity, allowing for spontaneous synaptic plasticity that can reshape network connectivity and enhance overall coordination of neural activity (Froese, 2015); psychedelics strongly evidence such effects (Carhart-Harris et al., 2014b; Gallimore, 2015).

Krippner (2000) characterized shamanic experiences as involving processes of deciphering images to infer meaning, engaging innate image-schemata to acquire information relevant to survival decisions. Hominin evolution involved an expansion of intentional ritualized alterations of consciousness that enhanced access to these internal experiences, propelling the emergence of the symbolic mind, and an acceleration of cultural evolution (Winkelman, 2002, 2009). Human cognitive evolution involved abilities to make symbolic interpretations of spontaneous images, using information acquired from normally unconscious processes for prediction of future conditions and responding to those challenges. Psychedelic alterations of consciousness enhance access to information that is normally unconscious, making it available through the visual symbolic processes that use image-schemas to integrate knowledge.

Another adaptive feature of psychedelic substances involves their contributions to enhanced social cohesion. Psilocybin produces a shift in emotional biases toward positive stimuli (Kraehenmann et al., 2016), decreasing visual threat processing by reducing the modulation caused by the top-down connections from the amygdala to primary visual cortex. Psilocybin reduces the role of the amygdala in tuning of visual regions in the face of threats, creating an acute shift in emotional valence toward an enhanced processing of stimuli with positive valence. This decrease of top-down connectivity in visual threat processing is accompanied by a shift in emotional biases toward positive interpretations. Similar dynamics are produced with LSD (Dolder et al., 2016; paraphrase p. 2638), which impairs recognition of sad and fearful faces; increases feelings of happiness, trust, closeness to others; produces enhanced explicit and implicit emotional empathy; increases participants' desire to be with others; and increases prosocial behavior. Psychedelic effects enhance the primary social functions of rituals by tuning interpersonal dynamics.

Pre-modern use of psychedelics involved an entheogenic view that these sacred plants contained spirit beings capable of producing changes in self, knowledge, personal powers and relations with others. Central to shamanic psychedelic experiences are encounters with a variety of spiritual entities, particularly animals, and the experience of a personal transformation into an animal form. Cross-culturally (Dobkin de Rios, 1984; also see Shanon, 2010; Winkelman, 2013b) the consumption of psychedelic plants is believed to release special powers inherent to these plants. Psychedelics are seen as producing experiences of the supernatural, including the signature experiences of altered consciousness in shamanism, the separation of one's soul or spirit from the body and its travel to the supernatural world. The intimate relationships of psychedelic plant use to shamanic rituals and spirit relations indicates the likelihood that use of psychedelic substances was part of the processes of selection for human cognitive sensitivities to engage these types of beliefs.

## Dis-inhibitory processes in the brain during altered consciousness

The fundamental similarities of psychedelic-induced and naturally-induced mystical experiences (Smith, 2000; Yaden et al., 2017) support the classic perennialist view of fundamental commonalities to mystical experiences across cultures and their independence of the mode of induction. These parallels point to the need for an explanation of the neuropharmacological effects of psychedelics in relationship to the non-drug mechanisms that elicit comparable experiences. Why do the same visionary dynamics of psychedelic experiences emerge in other contexts where drugs are not the causal factor?

The idea that diverse methods of altering consciousness produce similar experiences through eliciting similar brain responses has a long history. Mandell (1980) proposed a general physiological mechanism underlying transcendent states that is evoked by many different drugs, physiological conditions and procedures (i.e., hypnosis, meditation) which result in the loss of serotonin inhibition of the hippocampal cells. This loss of inhibition leads to an increase in cell activity and manifestation of hippocampal-septal slow-wave EEG activity that projects a synchronous slow-wave pattern across the lobes of the brain. Activities such as long-distance running, food and water deprivation, sleep loss and auditory stimuli such as drumming and chanting elicit a similar driving response in the brain (see Winkelman, 2013a for review). Mandell (1980) also proposed that the hippocampus is the focal point of the mechanisms that reduce the inhibitory serotonin regulation of temporal lobe limbic function, resulting in a reduction of the gating of emotional responses and an enhancement of activity in the dopaminergic circuitry. The loss of serotonin inhibition over various brain regions results in hypersynchronous discharges across the hippocampal-septal-reticular-raphe circuit.

This general dynamic of alteration of consciousness is typified by psychedelics. The phasic effects of psychedelics first stimulate and enhance serotonin; secondly, saturate and overload the serotonin system; and thirdly, release the habitual serotonin repression of the dopaminergic system (Passie et al., 2008; Previc, 2009). Psychedelics' resistance to normal reuptake mechanisms locks out serotonergic transmitter sites and saturates the serotonergic system, habituating the receptors and reducing the regulatory processes of the serotonergic system. This results in a release of the dopamine system normally repressed by serotonin. The reduction of serotonergic and noradrenergic modulation (control) results in the ascendance of the dopaminergic and acetylcholine systems that produce a variety of notable visual syndromes, especially hallucinations and dreaming (Hobson, 2001; Previc, 2009).

Psychedelic interruption of cortico-striato-thalamo-cortical loops that inhibit the lower brain structures' sensory gating systems (Vollenweider, 1998; Vollenweider and Geyer, 2001) results in enhanced availability of information normally repressed by these systems. Vollenweider (1998) attributed the mechanisms of action of psychedelics to effects on the frontal-subcortical circuits, principal organizational networks involving neuronal linkages and feedback loops of the frontal cortical areas with the thalamus. Psychedelic interruption of serotonergic inhibition of thalamic screening results in a flood of information that can overwhelm the frontal brain with a variety of normally repressed sensations that enhance the availability of information managed by these ancient levels of the brain.

The alteration of consciousness through disruption of the normal brain control activities can be achieved by diverse means, including psychological, physical and physiological stimulation and disease (Vaitl et al., 2005). Vaitl and colleagues attributed the general causes of alterations of consciousness to compromised brain structures, disconnectivity in brain dynamics between distributed brain regions, or imbalances in neurochemical and metabolic processes. Vaitl et al. (2005), noted that interruptions, asynchrony and uncoupling in thalamic systems produce various anomalies of consciousness. Many alterations of consciousness involve temporally disconnected oscillatory activity of the thalamocortical circuits or defective connectivity between distributed brain regions. This disconnectivity results in the emergence of activity in the ascending thalamocortical systems, producing hallucinatory experiences as a result of intrinsic brain activity, which is no longer as constrained in the absence of sensory input.

The alteration of consciousness through disruption of normal control processes was also proposed by Dietrich (2003), who reviewed evidence reporting that the deregulation of higher order functions of the prefrontal cortex was associated with endurance running, dreaming, hypnosis, drug induced states, and meditation. d'Aquili and Newberg (1999) proposed that the properties of mystical experiences result from processes like deregulation, a deafferentation of normal input, and a functional blocking of input into a brain structure. Enhanced attentional processes characteristic of meditation result in deafferentation of input from other systems, such as the environment, that would be distracting to highly focused attention. As a result of deafferentation, certain neural structures gain independence from normal input and instead start firing according to their own internal logic or pattern.

Barrett and Griffiths (2017) reviewed research on the similarities of meditation and psychedelics on brain systems, and proposed that their similarities involve effects on key areas of the DMN. These parallels in effects of psychedelics and meditation on key brain areas support the idea of common mechanisms underlying psychedelic-induced and other visionary experiences.

### Psychedelic disruption of the default mode network

A number of research projects beginning with Carhart-Harris et al. (2012) reported psychedelic effects involving a disruption of the DMN (see Table 1). The DMN involves a network of regions that function as hubs for the structural and functional connections that underlie a range of meta-cognitive processes that are normally activated during an inward focus of attention such as activities of introspection and daydreaming (Buckner et al., 2008; Uddin et al., 2009; Scheibnera et al., 2017). These forms of metacognition involve processes that underlie self-consciousness, reflective self-awareness and self-representation, as well as perceptions of social others. The DMN is also active in mental time travel, which engages autobiographical memory as a reflection on one's personal past; and projects one's self into the future, with imagined possible outcomes. Khachouf et al. (2013) noted that the varied phenomenal qualities associated with the ordinary functioning of the DMN engage in representations of scenarios different from what is actually going on in the immediate environment, a simulation of possible life circumstances; and that these imagined mental perspectives are in reference to one's self. These kinds of thought processes might intuitively seem like the activities underlying psychedelic experiences, but the effects of psychedelics disrupt these habitual patterns of thought.

**Table 1:** Psychedelic effects on the PFC and DMN.

Study:	Carhart-Harris et al., 2012	Kometer et al., 2015	Palhano-Fontes et al., 2015	Carhart-Harris et al., 2016
Substance:	Psilocybin	Psilocybin	Ayahuasca	LSD
PCC Activity	Decrease	Decrease	Decreased	Decrease
Connectivity			Reduced DMN, precuneus	Decrease vmPFC, PH/RSC
^*^mPFC Activity	Decrease		Decreased	Increase
Connectivity	Decrease PCC			Increased w/ caudate/
				Inf. Front. gyrus
AG Activity	Decrease			Increase of vmPFC/
Connectivity				Inf. Front. gyrus
^*^dmPFC Activity			Decreased	
Connectivity				
TPJ				
Activity				
Connectivity				
^*^LTC				
Activity				
Connectivity				
ATP Activity				
Connectivity				
^*^HP Activity				
Connectivity				
PH Activity		Decrease		Increase
Connectivity				Decreased RSC
				Increase dorsal mPFC
^*^RSC Activity	Decrease	Decrease		Decrease PH/PCC
Connectivity				
^*^pIPL Activity	Decreased PCC			
Connectivity				
^*^ACC Activity	Decrease	Decrease		
Connectivity				

* *, PFC component; PCC, Posterior cingulate cortex; mPFC, Medial prefrontal cortex; AG, Angular gyrus; dmPFC, Dorsal medial prefrontal cortex; TPJ, Temporoparietal junction; LTC, Lateral temporal cortex; ATP, Anterior temporal pole; HP, Hippocampus; PHC, Parahippocampus; RSC, Retrosplenial cortex; pIPL, Posterior inferior parietal lobe; ACC, Anterior cingulate cortex*.

Raichle (2015) characterized the DMN as based in the medial and lateral cortices of the parietal and temporal lobes, as well as medial prefrontal cortex (mPFC). Its major subdivisions include the ventral medial and dorsal medial regions of the prefrontal cortex, as well as the posterior cingulate cortex (PCC; with precuneus) and the lateral parietal cortex. The DMN primarily involves connections among the thalamus, PCC and mPFC and areas of the limbic system (parahippocampal cortex and the hippocampus) that function as a network for information routing and integration (Buckner et al., 2008). Raichle (2015) concluded that the cortico-cortical axonal pathways involve a structural core comprised of the posterior medial cortex and parietal cerebral cortex, as well as the temporal and frontal modules that link all major structural hubs of the brain through the posterior areas of the DMN, which provide central functional integration of the brain networks. The DMN is functionally and anatomically connected with the thalamus and precuneus, a connectivity that is crucial to consciousness (Cunningham et al., 2017).

Psychedelic stimulation of excitatory cortical neurons with serotonin 2A receptors disrupts the normal rhythmic oscillations of cortical neurons; this results in a decoupling between cells, leading to an overall disorganization of cortical activity (Carhart-Harris et al., 2014a). Psychedelics cause reduced activity in the DMN's hub structures, which leads to a reduction in oscillatory activity and network integrity (Carhart-Harris et al., 2012, 2016; Muthukumaraswamy et al., 2013; Palhano-Fontes et al., 2015; see dos Santos et al., 2016 for review). The overall result is reduced functional connectivity among areas of the DMN. In particular, decreased integrity of the DMN was reflected by a decreased functional connectivity between the parahippocampus and retrosplenial cortex, the magnitude of which was strongly correlated with self-report ratings of experiences of spiritual experience and “ego-dissolution” (Kometer et al., 2013; Lebedev et al., 2015; Carhart-Harris et al., 2016; Tagliazucchi et al., 2016). Muthukumaraswamy et al. (2013) also found that psilocybin caused a reduction in oscillatory activity and power in posterior and frontal association cortices and the DMN. This results from decreased functional coupling between the frontal cortex and the medial temporal lobe components of the DMN, as well as between the medial prefrontal cortex and the PCC.

A review (dos Santos et al., 2016) of 25 neuroimaging studies of serotonergic psychedelics concluded that a wide range of imaging studies show the excitatory effects of oral administration of psychedelics such as mescaline, psilocybin, and ayahuasca. There are effects in frontolateral/frontomedial cortex and medial temporal lobe areas that are known for their central roles in cognitive functioning, as well as self-awareness, emotional processing and memory. The review also revealed a decrease in functional connectivity between key hubs of the DMN as were reported in many studies (i.e., Carhart-Harris et al., 2012, 2016; Tagliazucchi et al., 2014, 2016; Lebedev et al., 2015; Palhano-Fontes et al., 2015; Kaelen et al., 2016); most reported a reduction in connectivity between the PCC and ACC.

There are some differences among the various studies of psychedelic effects on the DMN, reflecting issues such as which phase of the experience was studied (early, middle, vs. late), and the specific drug and dosage levels used. dos Santos et al. (2016), noted that some of these differences may be a result of the time-scales associated with the measurements used in different techniques; other differences may be due to yet-to-be-determined differences in the effects from oral vs. intravenous modes of drug administration. Studies on other 5HT2 psychedelic substances generally confirm this general effect on the DMN. Palhano-Fontes et al. (2015) found ayahuasca produced a significant decrease in activity throughout most parts of the DMN, especially in the hubs of the PCC, precuneus, and the mPFC. Ayahuasca also reduced functional connectivity within the PCC/precuneus.

### Psychedelic induction of bottom-up information transfer

In contrast to these acute effects of psychedelics in reducing functional connectivity within the DMN, there is an increased functional connectivity between normally disconnected brain networks. Psilocybin enhances novel connections among brain areas, manifested in increased connectivity between networks (Roseman et al., 2014; Kuypers et al., 2016) and a wider range of connectivity states (Tagliazucchi et al., 2014, 2016). Resting state networks showed a decreased functional segregation under psilocybin, forming especially stable functional connections that are present only during the psychedelic state (Roseman et al., 2014) and increasing communication across the entire brain (Petri et al., 2014). Psilocybin produces many new transient structures that are manifested in very strong, persistent topologically long-range functional connections that increase integration across cortical regions, resulting in an increased cross modular connectivity that produces more intercommunicative, less constrained, modes of brain function (Petri et al., 2014, p. 7, 8).

A change from top-down to bottom-up dynamics in the transfer of information under psychedelics is confirmed by Alonso et al. (2015), who assessed changes in directionality of information flow in the brain provoked by ayahuasca. Using measures of transfer entropy that assessed nonlinear changes in functional connectivity, they found significant changes in the connectivity of brain oscillations, particularly a disruption in the functional connectivity of brain oscillations that reflected modifications in the normal coupling between anterior and posterior areas of the brain. Their analyses of transfer entropy found decreases in the influence of frontal brain areas over the posterior areas, specifically in the central, parietal, and occipital regions. In contrast, there were increases in the influence of posterior regions on the frontal anterior brain regions. These complementary changes, anterior-to-posterior transfer entropy decreases, and their imbalance with the posterior-to-anterior transfer entropy were correlated with the intensity of subjective effects and degree of incapacitation reported by the participants. Psychedelics reduced DMN integrity and caused disintegration in normal system functions by reducing connectivity of the frontal cortex with lower brain areas (Alonso et al., 2015). “These results suggest that psychedelics induce a temporary disruption of neural hierarchies by reducing top-down control and increasing bottom-up information transfer in the human brain” (Alonso et al., 2015, p. 1).

General effects of psychedelics involve the temporary disruption of the normal neural hierarchy, replacing the normally predominant top-down control of information transfer in the brain with an increasingly bottom-up dynamic characterized by an increased influence of posterior regions over frontal areas of the brain (Riba et al., 2004). This decoupling of the frontal areas with the medial lobes resulted in a disorganization of the high-level networks responsible for large-scale brain network integrity, resulting in increased flexibility of networks and a more open communication among them. The decoupling between frontal and medial temporal lobe structures permits a freer operation of the latter, which is associated with more primary forms of consciousness based in a somatic awareness and subjective feeling states.

### Effects of other alterations of consciousness on the DMN

The interruption of the normal patterns of the DMN by psychedelics provides important information about the mechanisms of their neuropharmacological action in producing phenomenological experience, such as the relationship of the decreased connectivity between the parahippocampus and retrosplenial cortex in producing an experience of “ego-dissolution” (Muthukumaraswamy et al., 2013; Carhart-Harris et al., 2016). But these kinds of effects of reduced activity and connectivity within the DMN are also found with a number of non-pharmacological methods for altering consciousness. The interruption of ordinary control processes of the brain, which is caused by psychedelics, is also found with other consciousness altering practices such as meditation, hypnosis, and epilepsy. The commonalities involve compromising the integrity of the DMN, including its connectivity with other areas of the brain, especially the PFC. These similarities across methods for altering consciousness lead to specific hypotheses regarding the common mechanisms of visionary experiences found in association with psychedelics, as well as mysticism and other alterations of consciousness.

The major types of meditation techniques (Concentration, Loving-Kindness, and Choiceless Awareness) used by experienced meditators produce a relative deactivation in the main nodes of the DMN (Brewera et al., 2011). In all three mindfulness meditation conditions, key DMN regions (PCC and mPFC) showed less activity in meditators compared to the controls. Similar results were reported in an electroencephalography (EEG) and functional magnetic resonance imaging (fMRI) study (Panda et al., 2016) comparing 20 Raja Yoga expert meditators (“mental concentration”) with a control group to examine differences in the DMN. Their fMRI results showed that in comparison to the controls, the meditators had significant reductions in activity and connectivity of the DMN's posterior cingulate hub, which was not as strongly connected with other areas of the DMN. Another study (Scheibnera et al., 2017) of mindful attention practices found significantly less neural activation in two primary DMN hubs, the mPFC and PCC, as well as reduction in activity in the left temporoparietal junction. A meta-analysis (Fox et al., 2016) of 78 neuroimaging studies examining patterns of brain activation and deactivation associated with different styles of meditation found that focused attention meditation produced significant activation in two DMN areas (PFC and ACC), as well as a deactivation of two major DMN hubs, the PCC and the posterior inferior parietal lobule.

Hypnosis also reduces levels of activity in the anterior areas of the DMN (McGeown et al., 2009), with an association of the depth of attentional absorption induced by hypnosis with the degree of reduction of DMN activity (Deeley et al., 2012). High (vs. low) hypnotizibility subjects had greater decreases in DMN activity, particularly the dorsal ACC, and reduced connectivity of the PCC with areas of the Executive Control Network (Jiang et al., 2017).

The relevance of epileptic seizures to understanding psychedelic action on the brain is highlighted by many reports of ecstatic visionary experiences from people who appear to suffer epileptic attacks (Danielson et al., 2011). A wide range of brain abnormalities involving impairment of brain function frequently produced hallucinatory, self-transcendent and mystical experiences (Urgesi et al., 2010; Kastrup, 2017). A wide range of neuroimaging and electrophysiological studies report that epileptic seizures also impair the primary nodes of the DMN, particularly the precuneus/PCC, the medial frontal cortex and lateral parietal cortex, as well as the ACC (Danielson et al., 2011), with long-lasting decreases in DMN activity during and following seizures (complex partial, generalized tonic-clonic, and absence seizures). These outcomes suggest that decreases in DMN activity involve the inhibition of the subcortical arousal systems, a “network inhibition hypothesis” that seizure-induced inhibition of arousal systems causes deactivation in other cortical areas. Since the thalamus is strongly involved in all three seizure types (complex partial, generalized tonic-clonic, and absence seizures), the involvement of corticothalamic networks is implied (Danielson et al., 2011), and also implicated in psychedelic effects (Vollenweider and Geyer, 2001).

While this relationship of brain function impairment to visual experiences can be attributed to the effects of disruptions of inhibitory neural processes, Kastrup (2017) proposed that there are additional causal principles yet to be identified. Barrett and Griffiths (2017) noted the parallels revealed by neuroscience research in terms of the neural bases of psychedelic and meditative effects on the DMN. They reviewed research that supports the hypothesis that a key aspect of psychedelic and meditation-induced visionary experiences are a consequence of decreased activity and functional connectivity in the medial nodes of the DMN (PCC and MPFC), regions known to mediate aspects of self-referential processing that underlie mystical experiences of unity. Barrett and Griffiths (2017, p. 30) point to further similarities: “Decreased activity in the IPL (and specifically in the angular gyrus), and decreased communication between the IPL and other areas involved in maintaining a sense of self (such as the PCC) are observed both in studies of meditation and in studies of classic hallucinogens.”

The interruption of the DMN, particularly the PCC, by meditation, hypnosis and epilepsy, point to the necessity of explaining psychedelic effects in a context that provides a general explanation of the alteration of consciousness. But what produces this rich, intense, personal and sometimes overwhelming visual imagery?

## Hypotheses on the origins of visionary experiences: innate modules and operators

This need for a general explanation of psychedelic effects that includes these other methods of altering consciousness is emphasized by the fact that they are also associated with vivid visionary and hallucinatory experiences. What produces the resultant experiences? Destabilization of habitual cognitive mechanisms (i.e., DMN) has the effect of reducing top-down brain dynamics and facilitating a bottom-up information transfer. This permits the emergence of more ancient cognitive processing capacities of the brain, a release of innate modular systems of knowing that provide the commonalities that underlie these diverse methods for altering consciousness. The general hypothesis proposed here is that the visionary effects of psychedelics, as well as other mechanisms for altering consciousness, involve the release of innate intelligences, operators, and modules that provide the structure and content for these experiences.

The hypothesis that innate operators can explain features of psychedelic experiences is justified on several grounds: the evidence for innate modular functions in the entoptic images provoked by psychedelics and diverse conditions (Siegel, 1977); the modular structure of the human brain (Gardner, 1983, 2000); the explanations offered in the cognitive science of religion for supernatural experiences and beliefs in terms of the operation of innate modules (Boyer, 2001, 2017; Clements, 2017); and the relationship of an innate operator, the MNS, to the DMN and the expressive capacities of mimesis (Garrels, 2006, 2011).

### Entoptic forms as innate operators

The engagement of psychedelics with innate systems is exemplified in their stimulation of entoptic forms. Evidence that psychedelic substances stimulate the release of innate cognitive forms originated in Kluver (1928) studies of the effects of mescaline on subjective experiences. Reports of recurring geometric patterns led to the concept of entoptic images, visual experiences generated by mechanisms within the visual system. Kluver (1928) noted the recurrence of specific patterns that he labeled “form constants.” These were represented in several principal types: basic geometric forms and patterns; a lattice or grating, including honeycombs, checkerboards, and cobwebs; tunnels, funnels, and cones; spirals; and more complex phenomena based on combinations of these forms. Siegel (1977) found that the modifications of these basic forms and their transformation involved the inclusion within broader geometric patterns through incorporating the elemental forms within the borders of other figures; and repeated combinations of basic forms and their incorporation into ornamental designs and mosaics.

The biological bases for these form constants has been evidenced in their manifestation under diverse circumstances, not only a range of drugs, but also near-death experiences, hypnagogic states, medical conditions, sensory deprivation or overstimulation, flickering lights, and extensive chanting or rhythmic drumming (Siegel, 1977). The widespread manifestation of these visual elements under diverse circumstances attests to the elicitation of these innate mechanisms in producing these characteristic psychedelic experiences.

### Humans' innate intelligences: modules and operators

Evolutionary psychology (i.e., Barkow et al., 1992; Cummings and Allen, 1998; Carruthers and Chamberlain, 2000; Crawford and Krebs, 2008; Confer et al., 2010; Buss, 2015a,b) presents models of the evolution of a modular structure to the human brain that provide explanations of why humans so naturally experience supernatural entities. Evolutionary approaches propose a modularity of the human mind that resulted from the ancient acquisition of a number of adaptive innate capacities that have been called innate intelligences, modules and operators, each one dedicated to different specific cognitive functions (see Gardner, 1983, 2000; d'Aquili and Newberg, 1999; Ernandes, 2013). Ernandes distinguished cognitive operators from cognitive modules, with modules involving more specific functions and anatomical brain structures, while cognitive operators involve generalized functions that involve connections with many areas of the brain. The concept of modules also reflects the isolated functioning of process, while the concept of operators reflects the notion of automatic action in response to the appropriate triggering stimuli.

A range of findings support a view of the human mind as functioning through an integrated assembly of many functionally specialized modular psychological adaptations that operate mostly independently and unconsciously. Gardner's (2000) criteria for innate intelligences include: their isolated (dys)function because of brain damage; exceptional manifestation in idiot savants and child prodigies; an evolutionary plausibility; central core cognitive operations; a facility for encoding in symbol systems; a developmental history in their manifestation; and support from experimental and psychometric studies. Cognitive functions that are manifested cross-culturally point to their underlying biological dynamics involving neurognostic structures, the neurobiological structures of knowing that provide the universal aspects of the human brain/mind (Laughlin et al., 1992).

Gardner (2000; p. 57) characterize ten of these basic innate intelligences as biopsychosocial potentials inherent to our species:

1. an intrapersonal intelligence for looking in at one's own mind and the ability to use awareness of one's own capacities to regulate one's emotional life and relations with others;
2. an interpersonal intelligence, a capacity to engage a “theory of mind” to infer others' mental processes;
3. a linguistic intelligence (actually involves several capacities);
4. a logical-mathematical reasoning capacity that manifests in extreme forms in the idiot savants with superhuman math processing capacities;
5. a bodily-kinesthetic intelligence manifested in mimesis;
6. a musical intelligence to create and perform with sound and instruments;
7. a spatial intelligence for creating patterns in space;
8. a naturalist intelligence for species recognition and classification of species;
9. a spiritual intelligence providing a desire to know about non-material experiences and cosmic entities and engaging with spiritual, noetic and transcendent experiences; and
10. an existential intelligence that provides an ability to reflect cosmic issues.

It is apparent that not all of these modules are equally elicited by psychedelics or other alterations of consciousness, but many are. The features of these modules are prominently displayed in shamanic psychedelic experiences characterized by the entheogenic world view of human and animal-like spiritual entities with human-like cognitive and social properties and special powers derived from the psychedelic influences. Shamanic experiences also engage the bodily-kinesthetic intelligence manifested in dance and enactment; the naturalist intelligence involving the use of animal species for personal identity and power; and the use of musical intelligence, including chanting and drumming. Contemporary DMT experiences (Luke, 2011) emphasize spiritual entities that reflect the operation of the interpersonal intelligence, as well as spatial geometric visions, out-of-body experiences, mind-to-mind communication, and concerns with spiritual and existential matters.

Innate modules and operators are the result of adaptations made to solve specific problems typical of the hunter-gatherer lifestyle. Their manifestation under diverse influences reflects the elicitation of such adaptive responses. These include positive emotions such as happiness and bliss; although negative emotions may be involved in mystical and psychedelic experiences, they appear less central, perhaps because depression is not an adaptive response. The innate modules typically activated by psychedelics involve successful adaptations: agency detection, theory of mind/mind reading, animal intelligences, musical intelligence, mimetic enactment capacities, and others.

Ernandes proposes that while the frontal cortex becomes involved in the control of many innate operators, they mostly have their basis in the R-complex and its linkages with the limbic system since they are widely distributed in reptiles and/or mammals. This role of these ancient brain system attest to these modules as involving ancient adaptations. Psychedelic experiences should not be expected to manifest operators that depend on the higher level cognitive integration provided by the PFC or the self or autobiographical qualities maintained by the DMN. Psychedelics do not elicit mathematical intelligence, and certain language functions can be difficult if not impossible; a semantic function might notably remain while speech is generally compromised. In contrast Gardener's operators for intrapersonal, interpersonal, bodily-kinesthetic (mimetic), musical, and naturalist (animal) intelligence are prominently manifested in psychedelic experiences, especially in the form of supernatural entities.

### Innate intelligences and supernatural experiences

The idea that human beliefs in the spirit world result from basic brain operators is fundamental to the main theoretical perspectives in the cognitive science of religion (e.g., see Boyer, 2001, 2017; Ernandes, 2013; Clements, 2017). The human tendency to perceive animate entities wherever we look reflects the underlying roles of modular capacities. The ubiquitous human tendency to posit supernatural beliefs is a consequence of a Hyperactive Agent-Detection Device (HADD; Barrett, 2000), a tendency to perceive an active agent under ambiguous conditions. A fundamental function of the HADD is the attribution of the intentionality of an agent as a fundamental cause of any phenomena. Ernandes (2013) notes the ubiquity of this animistic response across animal kingdoms indicates the basis of the HADD is in reptilian and limbic operators. This agency operator or agent-detection function expanded across mammalian evolution through natural selection to enhance the capacity for detection of intruders and predators. This resulted in adaptations for the over-detection of agency because it has few costs in comparison to the high costs associated with the failure to detect predators.

These adaptive assumptions of an “unseen other” were in the case of humans augmented with other projective assumptions—the social other, the dominant other, the all-seeing other, and the mental states of these others. This “theory of mind” capacity to infer the mental states and intentions of others is extended in thinking about bodiless spirits. The significance of modules and operators in the explanation of religious behavior involves linking them together in ways that allows for the creation of a general model of a relationship among the elements of reality, particularly their temporal and causal relations, in order to develop explanatory models of the universe (Ernandes, 2013). This assumption of the operation of a causal operator contributes a sense of an unseen causative agent to account for the events perceived when the cause is unknown, providing a mechanism of supersensible forces and powers to fulfill human needs.

This capacity to detect something was expanded in humans through the modeling of the cognition of others via our own experiences, projecting our own dynamics for the interpretation of others. This engages an inevitable tendency to project our own internal psychodynamics as the framework for the interpretation of other's internal dynamics, and the basis from which the unknown becomes humanized with the projection of our own self, social and emotional qualities. We then come to perceive the world as human-like and project human-like capacities into the world in order to understand it through inference. This capacity to infer the mental contents of another's mind has an identified basis in an innate modular capacity expressed through the MNS.

### The mirror neuron system

Mirror neurons are brain cells that have the property of being activated when a person performs a particular motor movement, as well as when a person observes another person engaging in that same specific movement. In this respect the mirror neurons function both motor and sensory neurons, reflecting a common neural basis for both observation of and engagement in intentional and goal directed actions. The activation of mirror neurons by behaviors and images of behavior creates a shared experience through a common neurological system for the participant engaging in an action and an observer's experience of seeing that action performed (Garrels, 2006).

There are significant interactions between the MNS and the mPFC node of the DMN in mentalizing and embodied simulation processes (Molnar-Szakacs and Uddin, 2013). The ACC provides an important locus for the interaction of the DMN with the MNS (Uddin et al., 2007). Washington and VanMeter (2015) characterized the ACC/mPFC connectivity—the dorsal and ventral regions of the mPFC with the dorsal and ventral areas of the ACC—as constituting the most anterior area of functional connectivity within the DMN. The integrated cognitive and emotional functions of the dorsal and ventral ACC/mPFC and PCC make them the ideal components for the operation of a “life simulator,” engaging past experiences as a basis for exploration of anticipated social events and future scenarios (Washington and VanMeter, 2015). The strong connections of the ventral ACC to limbic structures (amygdala, hippocampus), cortical areas that mediate limbic structures (orbitofrontal cortex and anterior insula), and the periaqueductal gray, nucleus accumbens, and hypothalamus give it a central role in the assessment and mediation of emotionally salient stimuli (Washington and VanMeter, 2015).

This DMN connection with the MNS is stimulated by increased activation of right fronto-parietal regions, which overlap with MNS areas involved in the mapping of the actions of others via simulation within one's own motor system (Molnar-Szakacs and Uddin, 2013). The frontoparietal mirror-neuron areas provide a bridge between conceptualization of self and conceptualization of others through self-representation functions that are derived from co-opting the MNS system used to recognize the actions of others (Uddin et al., 2007). The imagination of self-produced activities or other's activities both activate the midline and frontoparietal structures; this suggests that imagination is the common representational domain for both DMN areas (cortical midline structures) and the MNS (Uddin et al., 2007, p. 156). The MNS appears to provide a lower level physical representational system that has important mental functions of imagination in social behavior, enabling us to mentally project events and simulate various outcomes instead of having to actually witness or participate in events. A basic function of the MNS is the active creation of internal representations of not only actions, but also the affects and motivations of others (Newlin, 2009); this makes it a primary candidate for the images experienced under psychedelic and other influences.

An amodal representational and conceptual system underlies the meaning of both language and images, a common system of semantic representation for comprehension of events whether the input modality is language (sentences) or images (pictures; Jouena et al., 2015). Brain activity manifested in conjunction with understanding sentences as well as pictures involves the same distributed cortico-striatal network that “included the middle and inferior frontal gyri, the parahippocampal-retrosplenial complex, the anterior and middle temporal gyri, the inferior parietal lobe in particular the temporo-parietal cortex [with]…a multi-component network reaching into the temporal pole, the ventral frontal pole and premotor cortex” (Jouena et al., 2015, p. 72). They note that this “meaning” network overlaps with major areas of the DMN and involves areas of the brain recognized for their role in “semantic memory, embodied simulation, and visuo-spatial scene representation” (Jouena et al., 2015, p. 72). This common system for understanding both verbal and visual events relies on a common distributed network that integrates perception, action and conceptual processing.

This form of visual thought is based in image schemas representing the basic structures of sensorimotor experience (Johnson, 1987), with the behavioral manifestations directly implicating the MNS. These sensorimotor structures derive from repeated patterns of interaction of the organism with the environment that are encoded in the most basic structures of the brain and body as external objects of perception. The image schemas that develop in our direct engagement with the world become the structures that represent our inner mental life and thought, a common structure for our relationships with the external world and our internal structures of experience. These imagined motor actions engage the same networks as the actual movements, providing a common basis for the interaction with the world and the re-imaging of those experiences symbolically.

The most fundamental schema for analogical transfer of meaning across domains involves the body's ability to act (Newton, 1996). It is an innate neurologically based body schema that provides a common basis of both somatic and symbolic levels of reality, allowing for visual information, behavioral information, and symbolic information to be manifested through the same medium (Winkelman, 2010a). It is this re-representation of information in different systems-visual, somatic, and behavioral—that provides the basis for symbolic thought that emerged first in the visual domain.

The functions of mirror neurons in representing visual images of others' behavior and our same behavior supports the hypothesis that mirror neurons mediate not only the production of internal visual representations, but visionary experiences as well. This visionary component of psychedelic experience ought to be measurable by the well-establish construct of Visionary Restructuralization (Dittrich, 1998; Studerus et al., 2010).

### Mimesis as the origin of human thought

The innate operation of a human cognitive processes known as mimesis is through the MNS (see Garrels, 2006, 2011 for review). The social functions of the MNS as an innate operator are illustrated by Ernandes (2013), who used MacLean's (1990) extensive study of ritualized behaviors to identify innate cognitive and behavioral operators. The mimetic operator is an extension of one of the most basic communicative operators, the isopraxic operator, which stimulates members of a species to perform the same actions that are observed in the behaviors of other members of the group. In humans this isopraxic operator of behavioral emulation of the “other” is extended in mimesis (see Gardner, 2000 bodily-kinesthetic intelligence). The mimetic operator engages bodily movements as symbolic communication devices, enhancing group coordination by building on the most basic animal display behaviors, repeating each other's behavior.

This preverbal communication system, manifested in bodily movements, facial expressions and gestures, is an innate system that links the minds of human infants with their social world from the beginning of life. Mimesis produces meaning through metaphor, using gesture and imitation in an enactment that involves a mapping of body actions onto an imagined context (Newton, 1996). Body metaphors express meanings through the ability to mediate between the sensory domains and the domains of meaning through analogical reasoning processes involving the body.

Imitation was a basis for human evolution and the development of culture in early hominins, beginning with the primordial roles of mimesis in human learning and shared intersubjective experience (Donald, 1991, 2006). Donald (2006, p. 16) proposed mimesis was at the basis of a co-evolution of our capacities for cognition and culture, providing a “a single neurocognitive adaptation…[for] mime, imitation, gesture, and the rehearsal of skill.” This intuitive form of communication provided by the MNS also facilitated interpretation of complex social situations and attributing meaning to others through this capacity to infer others' thoughts, intentions and desires.

These expressive manifestations of mimesis in dance, music, and ritual provided a publicly accessible system of meaning. These expressive capacities of mimesis provided the basis for the evolution of the symbolic human mind into what Donald (1991) called mimetic culture and mythic culture. Winkelman (2010a) has shown how this mimetic complex of dance, music, drumming and rhythmic enactment was the context within which the collective rituals of ancient hominins were transformed into the visionary rituals of shamanism. Psychedelic plant use enhanced the access to this visual system and its information capacities.

The recognition of these visual experiences of psychedelics as symbolic representations has a precedent in the concept of presentational symbolism (see Langer, 1951; Hunt, 1995). Presentational symbolism is an imagetic capacity that is foundational to meaning-making, a symbolic representation system that precedes and supports our rational, language based consciousness (see Winkelman, 2010a for discussion). This ancient mode of imaginal consciousness appears in dreams and daydreaming, as well as shamanic visions, mystical experiences, near-death experiences, out-of-body experiences, and psychedelic visions (Horváth et al., 2017). These processes may be released by a variety of mechanisms that cause disruptions in filtering processes that normally repress these archaic forms of cognition.

Horváth et al. (2017) proposed that these processes also function constantly in our daily life, a kind of autonomic cognitive act that produces conscious experiences and affect in a synthesis of perception and thinking. Visionary experiences express a personal affectivity and representational capacities that directly present to the subject material that emerges from their own deep personal affective layers of consciousness. Psychedelic-induced visionary experiences involve the stimulation of powerful manifestations of this latent human cognitive capacity that is a background to all experience. When released by psychedelics this visionary modality easily and effortlessly takes dominance of our consciousness through an internal engagement with deep narrative levels of the mind that present the significant affective dynamics of life. Lohmar (2016; p. 20) noted that this expressive system provides a medium for three forms of material essential for the performance of thinking: the ability to retain in mind an object; engender other cognitions regarding this image object; and manipulate these to consider future possibilities.

## Discussion: a neurophenomenology of psychedelic mystical experiences

The similarity in phenomenal experiences produced by psychedelics and other alterations of consciousness attest to the necessity of a biological model of their causation. While the direct evidence for the specifics of neuropharmacological causation of phenomenal experience is limited, there are bases for hypotheses about these underlying causal mechanisms. Simultaneous assessment of specific mystical experiences and brain connectivity dynamics awaits methodological advances, but correlations of the various scales of mystical experience and ASC with gross changes in brain connectivity during sessions suggests the likely underlying neurological mechanisms producing the phenomenal experiences. Given the limited studies examining psychedelic neurophenomenological dynamics, brain imaging studies of similar meditative experiences can also suggest hypotheses regarding the mechanisms by which psychedelics produce mystical experiences (see d'Aquili and Newberg, 1999, 2000).

Mystical effects induced by psychedelics are partly the consequence of inhibiting the normal functions of the DMN. Disruption of connections of the parahippocampus with the retrosplenial cortex is directly implicated in the experiences of ego dissolution and dissolution of self-other boundaries characteristic of mystical experiences (Kometer et al., 2013; Lebedev et al., 2015; Carhart-Harris et al., 2016; Tagliazucchi et al., 2016). However, it is not merely the absence of DMN integrity which produces these experiences. These egoless experiences as reflected in the measure of “oceanic boundlessness” are associated with alterations in the frontolimbic-parieto-striatal network and positively correlated with the cerebral glucose metabolism in various brain areas, including DMN regions (ACC, HP, inferior parietal cortex), and PFC areas (frontomedial superior, frontolateral, and left inferolateral; Vollenweider and Geyer, 2001).

Disrupted self functions may also underlie experiences of unity and a sense of connection with the universe made possible by the absence of a separating sense of self. The changes in experience of self and relationship to a spiritual “other” typical of personalized theistic mystical experiences likely involve consequences of this psychedelic induced disruption of DMN self-related functions. The high importance of a social “other” in some mystical experiences implicates the activation or disinhibition of areas of the dorsal medial subsystem of the DMN that manages social information.

Disruption of the DMN may also account for the frequently reported sense of ineffability—the inability to express the experience in words. The occurrence of increased meaningfulness under LSD (Preller and Vollenweider, 2016) is in notable contrast to the frequent difficulty of speech. I hypothesize that speech production is compromised because of the lack of connections with the neocortical components involving the frontal lobe (Broca's area), while the language capacity for understanding meaning persists because of its lower anatomical basis near the DMN component, the inferior parietal lobule in the superior temporal gyrus (Wernicke's area). This dynamic may explain the highly meaningful yet ineffable aspects of the experiences that are represented in a system of knowledge long recognized in philosophy and psychology in concepts such intuition, tacit and implicit knowledge.

Prominent features of mystical experiences involve happiness, joy, and bliss. Psychedelics both stimulate limbic areas that manage emotional information as well as compromise the serotonergic repression of the emotional brain, resulting in the release of normally repressed emotional dynamics. d'Aquili and Newberg (1999, 2000) proposed that the positive emotional experiences and profound quiescence of meditative practices result from a deafferentation of limbic stimulation from the peripheral parasympathetic system, combined with reverberating parietal lobule connections with PFC circuits that result in stimulation of both divisions of the Autonomic Nervous System and enhanced connections of the sensory association areas with the inferior parietal lobe, a key DMN area. Picard and Kurth (2014) provide a further hypothesis in their findings that intense feelings of bliss result from stimulation the anterior-dorsal insula.

The separation of the self from the body is a well-known mystical experience that is prominent in shamanic and psychedelic experiences; these experiences known as an out-of-body experience (OBE), soul flight and astral projection, involve a disintegration of the normal unity of the self, body and one's visual perspective of the world (Metzinger, 2009). OBE experiences involve an inner focus of awareness experienced as a separation of the self from the physical body, with the self experiences dominated by an internally generated visual field.

The neural correlates of these OBEs involve a disruption of a specific DMN area, the temporo-parietal junction (TPJ), which integrates bodily and sensory modalities (Lopez et al., 2008). Shamanic ritual practices disrupt this integrative function through the effects of excessive drumming and dancing (Winkelman, 2010a). The effects of extensive exercise overwhelm the vestibular system, resulting in habituation and a shutdown of the TPJ and sensory system. TPJ habituation results in a loss of functional connectivity with the motor and somatic functions and a dis-engagement of the self-image and awareness from the visual field and body. This allows for the experience of movement independent of the physical body. This disembodied conscious self which persists in the face of physical crisis provides experiences generally interpreted as justifying beliefs in a soul, spirits, and supernatural powers (Metzinger, 2009).

The importance of mental state and intentions on psychedelic effects is recognized in the notion that set and setting are the most important determinants of the specific qualities of psychedelic experiences. Differences of mystical and shamanic psychedelic experiences may reflect the shamanic practice of ingestion of psychedelics is conjunction with fasting, prolonged periods of dancing and extensive singing and chanting, factors which could be expected to alter the effects of the psychedelics on the brain.

### Commonality and diversity in psychedelic effects

5-HT2a has been considered the primary neurotransmitter system affected by psychedelics (Nichols, 2004, 2016; Hintzen and Passie, 2010), but action on other serotonin receptors and other receptor systems has also been established (Ray, 2010, 2012; Halbertstadt and Geyer, 2011). Additionally, LSD's hallucinogenic effect through binding with the 5-HT2A receptor can only occur when there is dimerization with the mGlu2 receptor (Rivero-Müller et al., 2013, p. 69). Consequently, 5HT2A receptor agonism is a necessary but not sufficient condition for psychedelic effects. Furthermore, there are diverse effects at the same receptor by similar agonists, and agonist action on the same receptor may activate different intercellular signaling pathways as a consequence of receptor oligomerization and receptor trafficking (Rivero-Müller et al., 2013).

Rolland et al. (2014) noted that hallucinations may be induced by a variety pharmacological mechanisms, including the hyperactivation of dopamine receptors, such as that caused by psychostimulants; stimulation of serotonin 5HT2a receptors targeted by psychedelics; and the blockage of glutamate NMDA receptors caused by dissociative anesthetics. They proposed that these different pharmacological systems might share common neurobiological pathways involving integrated neurobiological circuits that when compromised can produce hallucinations. And they hypothesized that diverse mechanisms, including dopamine and serotonin activation and NMDAR blockage, can disrupt the thalamic gating functions and cortico-striato-thalamo-cortical loops, resulting in a disorganization of the brains basic filtering processes.

While many psychedelics may share common effects, they also have distinctive effects, neurologically as well as phenomenologically (see Ray, 2010, 2016). In assessing the different profiles of neurotransmitter interactions by various types of psychedelics, Ray (2016, p. 49) noted that “[m]ost of the drugs studied interact with multiple receptors, and most of the receptors studied interact with multiple drugs” This leads Ray (2010, 2012) to challenge the dominant theory of psychedelic action as being primarily mediated by effects at the 5HT2 receptors. Examination of the relative affinity of various psychedelics for a wide range of receptors (Ray, 2010, p. 22/41) found that “LSD has the strongest interaction collectively with the five dopamine receptors…[and] DMT has the strongest interaction with any single dopamine receptor.

Previc (2009) also proposes that diverse alterations of consciousness are a function of the activation of the dopamine system, which is directly stimulated by many different neurotransmitters and drugs and indirectly through effects on other neurotransmitter systems. Previc (2006, 2009) notes that dopaminergic activation favors extrapersonal cognition over body-based cognitive processes, enabling context independent cognition that processes information about events distant in space and time and cognition about occurrences other than those in the immediate present, typified in the OBE. Previc reviews evidence indicating a common underlying mechanisms for diverse methods of altering consciousness involving a disinhibition of dopaminergic extrapersonal brain systems, particularly those in the ventral cortex and the limbic circuit. Independent of the specific neurotransmitters involved, diverse processes producing visionary experiences may share common underlying mechanisms in a thalamic sensory overload, the common pathway resulting in a disruption of cortico-subcortical processing (Vollenweider, 2001).

This common pathway can carry a variety of different features of experience produced by the distinctive qualities of the various psychedelic substances. Ray (2012, 2016) proposes that the diversity in the phenomenology of psychedelic experiences is a consequence of the distinctly different neurotransmitter receptor profiles that each substance engages. Each neurotransmitter system (i.e., the various serotonin receptor subtypes, beta receptors, dopamine, histamine-1, imidazoline-1, kappa, mu, sigma, and cannabinoid receptors) elicits a specific profile of effects that Ray calls a mental organ. Each psychedelic drug has effects on a range of neurotransmitter systems that results in its characteristic effects on neuronal activity and on consciousness.

## Conclusions

Similarities in visionary experiences across diverse modes of altering consciousness attest to a common mechanism released by the interruption of the PFC and DMN. The disruption of the top-down control of the brain normally mediated by the functions of the PFC, together with the compromised self-referencing processes of the DMN, leads to the emergence of processes that are normally repressed/regulated lower level brain systems These ancient brain systems are manifested as innate intelligences, modules and operators. Various findings support the hypothesis that one of these innate capacities, the mirror neurons and their operation in the mimetic capacity, is at the basis of psychedelic and other visionary experiences. This novel hypothesis regarding the MSN as a mechanisms of psychedelic visionary experiences is supported by evidence that psychedelics elicit innate brain functions (entoptics), and the roles of the MNS in the integration of visual experience and behavior and in providing a common basis for self and other perceptions.

This ancient visual modality of information presentation and knowing elicited by psychedelics has seldom been studied scientifically because of its inaccessibility to intersubjective examination. Unfortunately, no mechanism allows us to share visual experiences to the same extent that words allow us to share our thoughts. The neuropharmacological dynamics of psychedelics powerfully and reliably elicit this mode of symbolic operation, making this modality of consciousness directly and intensely accessible. The repeatable effects of psychedelics in producing such visions and mystical experiences make them an unparalleled tool for the examination of the operation of this cognitive-affective system that is an innate aspect of the human brain-mind. Psychedelics consequently can serve as tools to provoke and expose this system, facilitating the examination of an area of human knowledge that has remained marginalized because of its notoriously subjective qualities. Through the use of psychedelics we can come to better understand the nature of some of the ancient symbolic and conceptual capacities of the human brain and the kind of experiences that generate the human quest for transcendent knowledge and spirituality.

## Author contributions

The author confirms being the sole contributor of this work and approved it for publication.

### Conflict of interest statement

The author declares that the research was conducted in the absence of any commercial or financial relationships that could be construed as a potential conflict of interest.
